# Behavioural response of a migratory songbird to geographic variation in song and morphology

**DOI:** 10.1186/s12983-014-0085-6

**Published:** 2014-11-28

**Authors:** Kim G Mortega, Heiner Flinks, Barbara Helm

**Affiliations:** Department of Migration and Immuno-Ecology, Max Planck Institute for Ornithology, 78315 Radolfzell, Germany; Department of Ornithology, University of Konstanz, 78457 Konstanz, Germany; Am Kuhm 19, 46325 Borken, Germany; Institute of Biodiversity, Animal Health and Comparative Medicine, University of Glasgow, G12 8QQ Glasgow, UK

**Keywords:** Sexual selection, Population divergence, Reproductive isolation, Phenotypic traits, Geographic clines, Simulated territorial intrusion, *Saxicola torquata*, Songbird, Behavioural isolation barrier

## Abstract

**Introduction:**

Sexually selected traits contribute substantially to evolutionary diversification, for example by promoting assortative mating. The contributing traits and their relevance for reproductive isolation differ between species. In birds, sexually selected acoustic and visual signals often undergo geographic divergence. Clines in these phenotypes may be used by both sexes in the context of sexual selection and territoriality. The ways conspecifics respond to geographic variation in phenotypes can give insights to possible behavioural barriers, but these may depend on migratory behaviour. We studied a migratory songbird, the Stonechat, and tested its responsiveness to geographic variation in male song and morphology. The traits are acquired differently, with possible implications for population divergence. Song can evolve quickly through cultural transmission, and thus may contribute more to the establishment of geographic variation than inherited morphological traits. We first quantified the diversity of song traits from different populations. We then tested the responses of free-living Stonechats of both sexes to male phenotype with playbacks and decoys, representing local and foreign stimuli derived from a range of distances from the local population.

**Results:**

Both sexes discriminated consistently between stimuli from different populations, responding more strongly to acoustic and morphological traits of local than foreign stimuli. Time to approach increased, and time spent close to the stimuli and number of tail flips decreased consistently with geographic distance of the stimulus from the local population. Discriminatory response behaviour was more consistent for acoustic than for morphological traits. Song traits of the local population differed significantly from those of other populations.

**Conclusions:**

Evaluating an individual’s perception of geographic variation in sexually selected traits is a crucial first step for understanding reproductive isolation mechanisms. We have demonstrated that in both sexes of Stonechats the responsiveness to acoustic and visual signals decreased with increasing geographic distance of stimulus origin. These findings confirm consistent, fine discrimination for both learned song and inherited morphological traits in these migratory birds. Maintenance or further divergence in phenotypic traits could lead to assortative mating, reproductive isolation, and potentially speciation.

**Electronic supplementary material:**

The online version of this article (doi:10.1186/s12983-014-0085-6) contains supplementary material, which is available to authorized users.

## Introduction

Phenotypic traits involved in signalling, for example aspects of song and morphology, are known to contribute to reproductive isolation between diverging populations [1,2]. Specifically, signalling in the context of mate attraction or territoriality may promote reproductive isolation through assortative mating and settlement patterns [3-5]. In birds, both sexes can be actively involved in signalling and also in discrimination of local conspecifics as potential sexual partners or sexual competitors [6].

In most songbirds, songs are a key component of signalling and are culturally transmitted across generations via vocal learning [7]. Young birds learn to produce or recognize song early in life, while still in their natal region. The geographic variation of such song traits is thought to result from the effect of imperfect song copying [8]. Accordingly, song dialects, i.e. the unique repertoire of shared songs within a population, combined with female preference for a local dialect due to parental imprinting, may lead to reproductive divergence [9-12]. Female preference for familiar vocalizations has been shown in captive and field experiments by increased copulation-solicitation displays to standardized playback [13-16].

Often not only vocalizations but a suite of selected traits of different sensory modalities contribute to the establishment and maintenance of reproductive isolation [17]. For example, morphological traits are also proposed to facilitate pre-mating isolation barriers between related avian lineages [18]. Such traits often include plumage coloration, e.g. redness in house finches, *Carpodacus mexicanus* [19]. In golden-collared manakins, *Manacus ssp.,* the golden is preferred over the white phenotype [20]. Genetically inherited visual signals may therefore facilitate diversification [21,22]. In contrast, sexually selected traits that are inherited culturally, notably learned avian vocalizations, can change instantaneously without requiring genetic change. They may therefore be a more efficient mechanism for reproductive isolation than inherited traits [23-26].

By promoting isolation, geographically differentiated signals are thought to aid local adaptation. The local adaptation hypothesis predicts that birds which select mates from their natal regions will gain fitness advantages because their offspring will more likely express adaptations to local ecological conditions [27], for example adaptations of seasonal activities associated with local climates, or morphologies tailored to specific lifestyles [28,29]. In North American crossbills (*Loxia curvirostra* - complex) distinct song types are associated with incipient speciation [30-32]. Interestingly, the differences in song types are coupled with morphological differences relating to ecological speciation. However, the processes of local adaptation and associated signalling may be sensitive to movement behaviour [33]. Migration may counteract population divergence [34] because: a) migration is thought to correlate positively with dispersal distance, which in turn generally promotes gene exchange [33,35]; b) migrants are typically under pressure to make rapid reproductive decisions, implying that female migrants may be less choosy than female residents [36], and may therefore not pair with the best (i.e., locally adapted) mate available [37]; c) relating to acoustic signals, migratory departure after breeding limits opportunities for young males and females to learn or imprint to the local dialect. Earlier studies have reported lower song discrimination in migrant than resident species, but have also indicated mechanisms by which migrants could nonetheless learn local song dialects after dispersal [34,38].

To better understand processes of local differentiation, in particular in migratory birds, we investigated discriminatory abilities in Stonechats (*Saxicola torquata* and closely related lineages [39]). The *Saxicola* complex has a wide distribution range, comprising substantial local differentiation in seasonal and morphological traits [40]. We focused on the short-distance migrant European stonechat (*Saxicola torquata*), which is socially monogamous with seasonal pair bonds selected by females [41]. During the entire breeding season, males defend their territory with distinct behavioural responses. Females also actively respond to conspecific intruders [42]. The fact that males sometimes “punish” their mates for their response to intruders indicates a sexual context to female interest [43]. The female responsiveness allowed us to examine discriminatory abilities in both sexes. We studied song variation between Stonechat populations and tested the behavioural response of the focal European population to song recordings and stuffed decoys. Early in the breeding season we obtained and analysed song repertoires of the local population and additional populations that breed 90 and 180 km away. We experimentally tested the responsiveness of local Stonechats to song from these populations and to stimuli from African Stonechats and a control species by conducting simulated territorial intrusions with playbacks. We also conducted a decoy experiment simulating a territorial intrusion by presenting a taxidermic mount of phenotypes from populations with differing geographic distances. The experiments focused on male response, but we also report data on the latency of the female response to the stimuli. All experiments were conducted during the breeding season at defined breeding stages in the presence of both pair mates.

In view of the geographic differentiation within Stonechats, we hypothesised that despite their migratory behaviour female and male Stonechats i) can discriminate between phenotypes of geographically distinct populations during playback and decoy experiments, ii) respond most strongly to local population stimuli, and iii) may show a consistent decline in their responsiveness with geographic distance. Furthermore, we hypothesised that songs may elicit stronger responses than morphological traits in both sexes because they may have diverged more rapidly.

## Results

### Song traits

**Figure 1:**
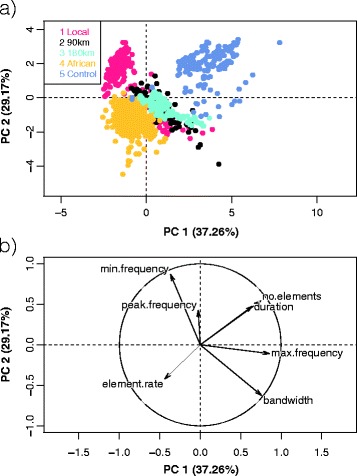
**Geographic variation in the song of stonechats and a control species as quantified by principal component analysis.** Shown is **(a)** the variation in song structure of European Stonechats from (1) the local population, (2) a population from 90 km distance, (3) a population from 180 km distance, (4) African stonechats, and (5) control species (Winter wren) based on a principal component analysis (for details, see Table 1); and **(b)** factor loadings of the two first principle components for song duration, number of elements, element rate, the minimum, maximum and peak frequency, and the bandwidth. The arrow length indicates the degree, the arrow direction the association of factor loadings with the principal components PC 1 and PC 2.

The Stonechat populations differed in their song traits from each other. A principal component analysis of seven traits (Table 1a) showed that several principal components explained the variation in song (PC1 = 37.26, PC2 = 29.17, PC3 = 21.62, Figure 1). Based on the first principal component, the focal population differed significantly from the neighbouring population (90 km). Differences increased further with geographic distance from the local population (Additional file 1: Table S1, Figure S1), although Stonechats from 90 km and 180 km were not significantly different from each other (Table 1b, Figure 1).

**Table 1:** **Song traits**

**(a)**				
	**PC 1**	**PC 2**	**PC 3**	
Song duration	0.40	0.33	0.46	
No. of elements	0.46	0.38	0.29	
Element rate	−0.27	−0.29	0.46	
Peak frequency	−0.02	0.30	−0.63	
Min. frequency	−0.23	0.61	−0.04	
Max. frequency	0.53	−0.08	−0.27	
Bandwidth	0.47	−0.44	−0.14	
Eigenvalue	2.61	2.04	1.51	
% variance	37.26	29.17	21.62	
**(b)**				
**Fixed effects**	**estimate**	**s.e.m**	**t**	**p**
**Intercept**	−1.76	0.12	**−14.89**	**<0.001**
**90 km**	2.28	0.29	**7.97**	**<0.001**
**180 km**	2.38	0.18	**13.52**	**<0.001**
**African**	0.64	0.19	**3.44**	**<0.001**
**Control**	4.79	0.19	**25.45**	**<0.001**

(a) Factor loadings of the principal component analysis for seven song traits of European Stonechats from the local population, a population from 90 km distance, a population from 180 km distance, African stonechats, and the winter wren. (b) Results of general linear model testing whether the first principal component (PC1) differed between songs from different locations, estimated by maximum likelihood methods. Estimates for the different song locations refer to differences from the intercept estimate, which represents song traits of the local population. Subjects were included as random intercepts to control for repeated measures. ‘Significant’ differences are shown in bold.

### Playback and decoy experiments

**Figure 2:**
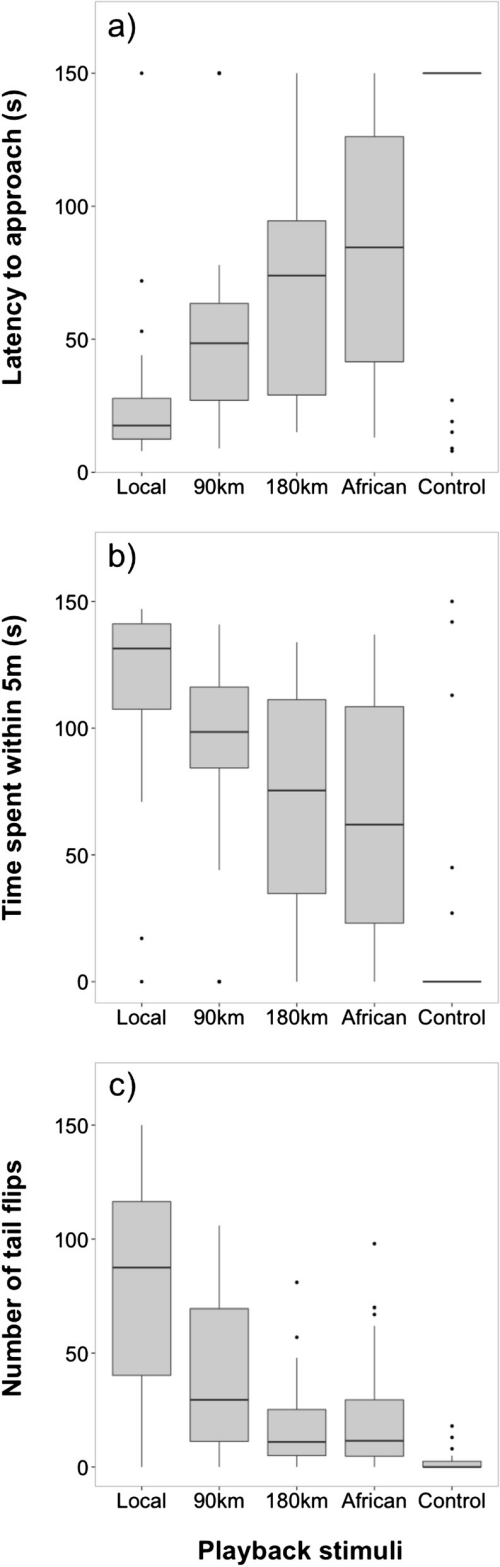
**Playback experiment in males.** Behavioural response for **(a)** latency to approach, **(b)** time spent within 5 m, and **(c)** number of tail flips in response to European Stonechats from (1) the local population, (2) a population from 90 km distance, (3) a population from 180 km distance, (4) African stonechats, and (5) control stimuli (Winter wren). Males discriminated between local and foreign stimuli by responding more strongly to song of their own population. Box plots represent, from bottom to top: minimum, lower quartile, median, upper quartile and maximum. Dots indicate observations further than one s.d. away from the mean; n =28.

Stonechats of the local population responded differently to stimuli from distinct populations, measured by the time they took to approach the caller or decoy (i.e., latency to approach within 5 m). In response to playback, males discriminated significantly between origins of the stimulus (z = −8.42, p <0.001, Table 2a, Figure 2). The males’ latency to approach the caller was lowest when exposed to the local song and increased with distance of stimulus origin (Table 2a, Figure 2). Breeding stage, trial order (Additional file 1: Figure S4), date and time of day showed no significant effect on the males’ latency to approach (Table 2a).

**Table 2:** **Latency to approach within 5 m**

	**Fixed effects**	**Estimate**	**Hazard ratio**	**s.e.m**	**z**	**p**
**Playback**						
(a) males	**Origin**	−0.86	0.42	0.10	−8.42	**<0.001**
	**90 km**	−1.30	0.27	0.31	−4.17	**<0.001**
	**180 km**	−2.17	0.11	0.34	−6.36	**<0.001**
	**African**	−2.32	0.10	0.35	−6.66	**<0.001**
	**Control**	−4.18	0.02	0.53	−7.85	**<0.001**
	Breeding stage	0.60	1.82	0.59	1.02	0.31
	Trial order	0.04	1.04	0.07	0.52	0.61
	Date	0.08	1.09	0.08	1.06	0.29
	Time	0.004	1.00	0.03	0.16	0.88
(b) females	**Origin**	−1.36	0.26	0.22	−6.28	**<0.001**
	**90 km**	−2.11	0.12	0.49	−4.32	**<0.001**
	**180 km**	−2.40	0.09	0.54	−4.45	**<0.001**
	**African**	−4.51	0.01	0.85	−5.32	**<0.001**
	**Control**	−6.88	0.009	0.97	−7.84	**<0.001**
	Breeding stage	−0.75	0.47	0.41	−1.84	0.06
	Trial order	0.16	1.18	1.27	1.29	0.20
	Date	0.02	1.02	0.07	0.23	0.82
	Time	−0.09	0.91	0.06	−1.60	0.11
**Decoy**						
(c) males	**Origin**	−0.78	0.46	0.16	−4.93	**<0.001**
	European	−0.43	0.65	0.41	−1.03	0.30
	African	−0.65	0.52	0.43	−1.52	0.13
	**Control**	−2.95	0.05	0.64	−4.62	**<0.001**
	Breeding stage	0.08	1.08	0.17	0.46	0.65
	Trial order	−0.18	0.83	0.16	−1.18	0.24
	Date	−0.05	0.95	0.11	−0.49	0.62
	Time	−0.001	0.99	0.009	−0.19	0.85
(d) females	**Origin**	−0.79	0.45	0.16	−4.84	**<0.001**
	**European**	−1.50	0.22	0.56	−2.65	**0.007**
	**African**	−1.70	0.18	0.59	−2.87	**0.004**
	**Control**	−3.19	0.04	0.84	−3.78	**<0.001**
	Breeding stage	−0.10	0.10	0.18	−0.54	0.59
	Trial order	−0.10	0.10	0.16	−0.59	0.55
	Date	−0.15	0.86	0.10	−1.44	0.15
	Time	−0.01	0.99	0.009	−1.50	0.13

Results of cox mixed-effects model with estimates, hazard ratio, standard error, z-value, and p-value fitted by maximum likelihood for playback in (a) males and (b) females, and decoy experiment in (c) males and (d) females. Estimates refer to differences from the intercept estimate, which represents the latency to approach of the local population (not shown). ‘Origin’ represents the overall estimate of differences between populations. ‘Significant’ differences are shown in bold.

**Figure 3:**
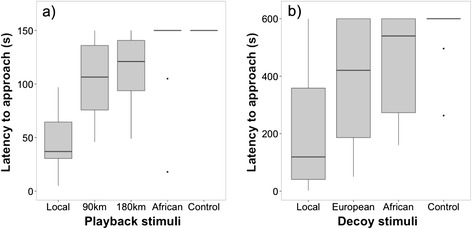
**Playback and decoy experiment in females.** Behavioural response for the latency to approach within 5 m in **(a)** playback experiments in response to song of European Stonechats from (1) the local population, (2) a population from 90 km distance, (3) a population from 180 km distance, (4) African stonechats, and (5) control stimuli (Winter wren); and **(b)** decoy experiments in response to stimuli from (1) local, (2) European, (3) African, and (4) control stimuli (European Robin). Females distinguished between stimuli by approaching the local stimuli significantly faster than all other stimuli. Detailed description of boxplots as in Figure 2; n =15.

Likewise, females also differed significantly in their behavioural response to different playback stimuli (z = −6.28 p <0.001, Table 2b, Figure 3). The females’ latency to approach the caller was lowest when presented with the local song and increased with geographic distance of the stimulus origin (Table 2b, Figure 3). Breeding stage, trial order, date and time had no significant effects on the females’ latency to approach the caller (Table 2b).

**Figure 4:**
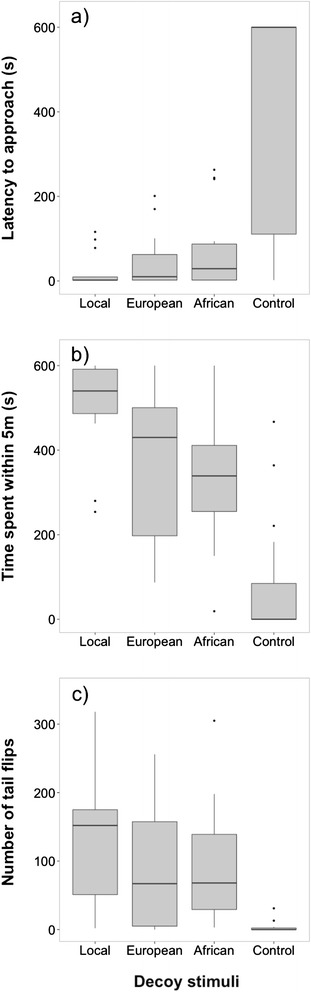
**Decoy experiment in males**. Behavioural response for **(a)** latency to approach, **(b)** time spent within 5 m, and **(c)** number of tail flips to (1) local, (2) European, (3) African, and (4) control stimuli (European robin). Males discriminated between local and foreign stimuli by responding more strongly to decoys of their own population. Detailed description of boxplots as in Figure 2; n =16.

During the decoy experiment, the males’ discrimination was less consistent than during the playback experiment (z = −4.93, p <0,001, Table 2c, Figure 4). The latency to approach local decoy stimuli did not differ from other European (z = −1.03, p =0.30, Table 2c, Figure 4) and African stimuli (z = −1.52, p =0.13, Table 2c, Figure 4), but males approached the control decoy significantly later than all others (z = −4.62, p <0.001, Table 2c, Figure 4). Breeding stage, trial order, date and time had no significant effect on the latency to approach (Table 2c).

Females showed finer discrimination (z =4.84, p <0.001, Table 2d, Figure 3). They approached the local decoy with lower latency than decoys of populations from greater geographic distances (Table 2d, Figure 3). Breeding stage, trial order, date and time showed no significant effect on the females’ latency to approach the decoy (Table 2d).

**Figure 5:**
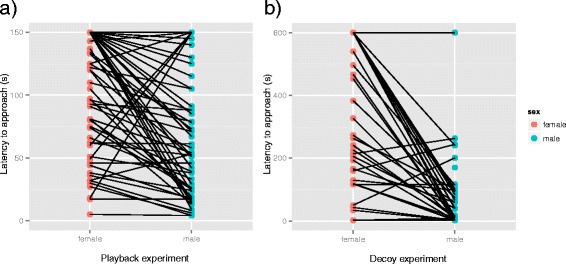
**Response behaviour of female and male mates within pairs.** Shown is the latency to approach within 5 m for females (red) and males (blue) within pairs (connected dots) for **(a)** the playback (n =15 pairs) and **(b)** decoy experiments (n =14 pairs) for all stimuli trials. Females and males differ significantly in their response behaviour, i.e. males approached the presented stimulus with lower latency.

There were no significant differences between different pairs, neither during the playback (z = −0.54, p =0.59, Additional file 1: Table S2a, Figure 5) nor the decoy experiments (z = −0.39, p =0.70, Additional file 1: Table S2b, Figure 5). Males approached the stimuli with significantly lower latency than females during both, the playback (z =4.78, p <0.001, Additional file 1: Table S2a, Figure 5) and decoy experiment (z =5.88, p <0.001, Additional file 1: Figure S2b, Figure 5). Breeding stage, date and time did not influence the response patterns of pairs (Additional file 1: Table S2). Trial order had no influence on the behavioural response of pairs during the playback, and only a slight but significant effect during the decoy experiment (Additional file 1: Table S2). Birds tended to approach the stimulus with lower latency in the first two compared to later trials (Additional file 1: Figure S4). A Spearman’s correlation test was run to determine the relationship between the behavioural response of female and male mates within a pair. The latency to approach was correlated between females and males during the playback experiment (r_s_ =0.51, p <0.001, n =15, Additional file 1: Table S3, Figure S2), but not during the decoy experiment (r_s_ =0.23, p <0.103, n =14, Additional file 1: Table S3, Figure S2).

Further behaviours of males also differed in response to stimuli from different populations. Males remained significantly longer within 5 m of the stimulus of the local population than of all other populations during the playback (Table 3a, Figure 2) and decoy experiment (Table 3b, Figure 4). Males of the local population also discriminated between origin of the stimuli in the number of tail flips, an indicator of agitation. In response to playback, the number of tail flips differed significantly between stimulus origins (estimate =16.65, t = −10.58, CI = −19.82, 13.45, Table 4a, Figure 2). The number of tail flips was highest when males were exposed to the local song and decreased with geographic distance of stimulus origin (Table 4a, Figure 2). Breeding stage, trial order, date and time showed no significant effect on the number of tail flips (Table 4a). Similarly, males also differed significantly in their number of tail flips during the presentation of decoy stimuli (estimate = −18.43, t = −1.30, CI = −48.35, 11.16, Table 4b, Figure 4). During trials of the local stimuli, males significantly flipped their tails more often than during all other trials (Table 4b, Figure 4). Breeding stage, trial order, date and time showed no significant effect on the number of tail flips (Table 4b).

**Table 3:** **Time spent within 5 m of the stimulus**

	**Fixed effects**	**Estimate**	**s.d.**	**CI 2.50%**	**CI 97.50%**
**(a) playback**	local	0.84	0.24	0.37	1.29
	**90 km**	0.18	0.21	−0.22	0.62
	**180 km**	−0.28	0.21	−0.70	0.14
	**African**	−0.37	0.22	−0.80	0.05
	**control**	−1.21	0.31	−1.82	−0.63
**(b) decoy**	local	−1.93	0.45	−2.75	−0.97
	**European**	−2.33	0.38	−3.03	−1.55
	**African**	−1.97	0.36	−2.65	−1.24
	**control**	0.59	0.36	−0.14	1.29

Results of generalized linear mixed model with estimates, standard deviation, and credible intervals using WinBUGS for (a) playback and (b) decoy experiment. Stimulus is defined as random factor to compare paths of all stimuli and thus correct for multiple testing. A behavioural response differs significantly from the local population if its credible intervals do not include the mean of the local population. Significant results are shown in bold.

**Table 4:** **Number of tail flips**

	**Fixed effects**	**Estimate**	**s.e.m**	**t**	**CI 2.50%**	**CI 97.50%**
**(a) playback**	stimulus	−16.65	1.57	−10.58	−19.82	−13.45
	local	21.34	25.35	0.84	−30.85	73.32
	**90 km**	−37.68	6.18	−6.09	−49.77	−25.20
	**180 km**	−57.62	6.16	−9.35	−70.01	−45.05
	**African**	−54.67	6.17	−8.86	−67.06	−42.01
	**control**	−74.16	6.46	−11.48	−87.36	−60.74
	breeding stage	23.13	10.37	2.23	1.93	44.08
	trial order	−3.10	1.44	−2.16	−5.97	0.44
	date	2.75	1.45	1.89	−0.18	5.69
	time	0.49	0.50	0.97	−0.54	1.51
**(b) decoy**	stimulus	−18.43	14.13	−1.30	−48.35	11.16
	local	91.14	75.87	0.80	−102.65	224.57
	**European**	−10.80	43.36	−0.25	−106.25	83.18
	**African**	−52.38	43.82	−1.20	−146.92	40.28
	**control**	−48.50	44.02	−1.10	−144.07	46.80
	breeding stage	−2.78	20.12	−0.14	−46.04	39.67
	trial order	59.11	16.39	3.61	24.44	93.31
	date	−15.99	11.76	−1.36	−41.19	8.33
	time	−0.28	1.00	−0.28	−2.40	1.82

Results of general linear mixed model with estimates, standard error, t-value, and credible intervals fitted by maximum likelihood. Estimates for the stimulus locations refer to differences from the intercept estimates, which represent the number of tail flips of the local population. Subjects were included as random intercepts to control for repeated measures. A behavioural response differs significantly from the local population if its credible intervals do not include the mean of the local population. Significant results are shown in bold.

## Discussion

This study reports clear differentiation in song traits of migratory European Stonechats over relatively short distances (90 km and 180 km from the focal population). By testing the behavioural responses to acoustic and morphological stimuli, we have also demonstrated the Stonechats’ ability to discriminate between geographic origins of sexually selected traits in two modalities. The responses of both sexes during playback and decoy experiments were graded and declined with increasing geographic distance from the local population. The concordance of these responses and the significant preference for the closest population suggests potential for the evolution of reproductive isolation although at present this is confirmed only for a single population.

Male and female Stonechats were similar in their behavioural discrimination, in contrast to results from other species. A recent study on Rufous-collared sparrows, *Zonotrichia capensis,* also reported discrimination between local and foreign stimuli, but the sexes differed in behaviour [16]. Females were presented with songs of the local, nearby nonlocal, and distant nonlocal dialect, and a control song from another bird species. They preferred the males’ local song dialect to all other dialects tested, showing significantly more copulation solicitation displays. In contrast, males displayed only a low discrimination ability between dialects of geographically close populations [16]. Similarly, in White-crowned sparrows, *Zonotrichia leucophrys*, females were more sensitive to geographic variation in song than males [44]. A study on hybridizing Pied flycatcher, *Ficedula hypoleuca*, and Collared flycatcher, *Ficedula albicollis*, revealed that females quickly recognise male species identity by song and have a strong preference for conspecific males resulting in assortative mating, and thus preventing further hybridisation [45,46]. In contrast, males of both species courted the heterospecific female and the conspecific female with similar intensity, thereby promoting hybridisation. This lack of species recognition could be due to mating being less costly in males, which can inseminate several females over a short period, while females are constrained by the number of their eggs. Females, therefore, should not make mistakes in mate choice [37]. The fine discrimination ability of Stonechats indicates that females may mate assortatively, while males may use the fine discrimination to fight off particularly attractive sexual competitors with local dialects. We cannot disentangle male and female responses because we conducted simulated territorial intrusions in presence of both pair members. An influence of the mate is suggested by the correlation between mates during the playback experiment (Additional file 1: Table S3, Figure 5) and has been shown previously in Stonechats [42]. Therefore, a crucial future step for a better understanding of the response to acoustic and morphological traits in Stonechats is to conduct experiments separately on females and males.

The local differentiation and consistent behavioural discrimination of song by origin of Stonechats, which migrate, was similar to that of resident species (e.g., indigobird *Vidua sp.* [47], Galapagos Sharp-beaked ground finch, *Geospiza difficilis* [6], and song sparrow, *Melospiza melodia* [24]), but differed from findings in some migratory species. Among *Zonotrichia* sparrows, long distance migrants (e.g., *Z. l. gambelii)* do not form dialects [34], whereas in sedentary *Zonotrichia* subspecies (e.g., *Z. l. nutalli)* geographic song variation occurs [13,15]. The corresponding lack of genetic diversification in *Zonotrichia* migrants, in contrast to significant genetic structuring among dialect areas in non-migrants, supports the idea that migration may counteract population divergence and isolation [48-50].

Although the fine acoustic discrimination ability of Stonechats suggests potential behavioural barriers, its implications for geographic isolation are not fully clear, partly depending on song plasticity, and ultimately on the mechanisms involved in song learning. In passerine birds, song is typically learned during a sensitive period early in life. In species like Stonechats that show geographic discrimination, males that subsequently disperse into ranges of other populations would face reduced mating prospects if an acoustic signature of the natal population remains in their repertoire [40]. However, this could be offset if the males were able to learn new songs after the sensitive phase. For example, migratory nightingales were able to acquire new song types in their first singing season [51,52]. In some species plastic song is based on an initial overproduction of learned songs during ontogeny [53]. Such overproduction of learned songs has been suggested to be correlated with a migratory lifestyle [38,54]. If present in Stonechats, plastic song learning could therefore enable dispersing males to be sexually selected by local females, although benefits of local song in sexual selection could be partly offset by the greater aversive response of local males. Dispersing females, in turn, may have no choice but to mate with a male singing a foreign dialect, and this might reduce population divergence. A modelling study by Ellers and Slabbekoorn suggests that evolutionary implications of song dialects are not straightforward [55]. Although in the majority of scenarios genetic and vocal divergence were concordant, the type of song learning and intrasexual competition in males affected the evolutionary outcome. For Stonechats, to answer this question unambiguously would require population genetic analyses alongside analyses of song traits among populations [56].

In our study, we found that Stonechats were also able to discriminate by morphological traits. Most studies of sexual selection do not explicitly test the role of simultaneous signalling with different sensory modalities, and instead focus on a single divergent signal or a suite of signals of the same modality [57-59]. In contrast, explicitly testing for effects of multiple signals enables the detection of divergent signal use in discrimination [60,61]. In Stonechats, we expected that culturally transmitted song may evolve more quickly, and thus could play a more important role for geographic clines than do morphological traits. We found that discrimination by song was more consistent than by morphological traits. The discrimination by song was sensitive to a geographic distance of only 90 km, whereas the decoy against which the birds visually discriminated originated from a population which breeds 1,000 km away. A caveat in the interpretation of these differences are the different breeding stages during which the stimuli were tested: song stimuli were applied during egg-laying and incubation stages, when birds may be particularly responsive, whereas decoys were tested during nestling and fledgling stages. However, Stonechats are multi-brooded, and females may initiate additional clutches while males take care of fledglings, so that male intruders may well gain reproductive benefits at this time. Moreover, in a study on closely related African stonechats with similar experimental designs, but conducted during simultaneous breeding stages for playback and decoy experiments, the birds’ discrimination by song was more consistent than that by morphology (unpublished data by KGM). Overall, our data cautiously suggest that song may be indeed the stronger discriminatory signal for Stonechats.

In the chestnut-bellied flycatcher, *Monarcha castaneiventris,* plumage colour played a greater role than song for the intensity of aggressive response by territory-owners, although both signals mattered [62]. Chestnut-bellied flycatchers display more variation in plumage colour than in song, which may indicate that plumage is more emphasised in sexual selection than song structure. The relative advantages of signalling with several modalities may be driven by the environment [63]. In general, acoustic signals can be transmitted over long distances and are ideal for long-range communication, whereas visual signals can be more limited and therefore be more suitable for short-range communication [64]. For Stonechats, which breed in open habitats, both signalling modes may be important.

## Conclusions

Our study on Stonechats reveals geographic differentiation of sexually selected traits in a migratory songbird. Song traits differed significantly in populations of distinct geographic origin. Consistently, both sexes distinguished local morphological and especially acoustic phenotypes from those of foreign populations. These data demonstrate that variation in sexually selected traits of different modalities may contribute to geographic isolation over relatively short distances, and thereby aid local adaptation. The sexes had similar sensitivity to incipient behavioural barriers. Maintenance or further divergence in these phenotypic traits could lead to assortative mating, reproductive isolation, and potentially speciation, in migratory Stonechats.

## Materials and methods

### Subjects

Stonechats inhabit open habitats across a large extent of the Palearctic [65]. The study population of European stonechats, *Saxicola torquata rubicola*, is located in northwest Germany (51°N, 6°30’E) and overwinters in the Mediterranean region, predominantly in north Africa [40,66]. The study population has been observed, measured and colour-banded for individual recognition since 1976. Stonechats arrive at the breeding grounds early in spring (late February/March), establish a territory, and form seasonal pair bonds with two to three broods per season [40]. After the postnuptial moult they start migrating towards the wintering grounds in early autumn [41,67]. In the present study, all focal pairs were ringed. We conducted regular checks twice per week to monitor the breeding activity and to define the breeding stage of each pair.

To test the discrimination ability of the local population, we collected songs from the local population (Düffel) and two nearby European Stonechat populations at distances of 90 km (Heubach) and 180 km (Wahner Heide) from the study area. Furthermore, we used songs and decoys of African stonechats from Kenya [distance 4,000 km, [68]] and decoys from Stonechats from Austria [distance 1,000 km, 41]. Control species are explained below.

### Recording method and song analysis

Stonechats, in common with most passerines, spend a higher proportion of their time singing just before dawn than at other times of day [69]. During the onset of the breeding season, we recorded the dawn song of a minimum of 28 individuals from each European stonechat population (n =3) for about ten minutes, using a Marantz PMD 661 solid state recorder (Osnabrück, Germany) and Sennheiser ME66/K6 directional microphones with windbreak (Georgsmarienhütte, Germany). To expand our set of stimuli, we also obtained 28 songs per species from African stonechats, *Saxicola torquata axillaris*, and Winter wrens, *Troglodytes hiemalis,* from the Macaulay Library (www.macaulaylibrary.org). These song recordings were conducted in the Great Rift Valley (Kenya) for African stonechats and New York State (United States) for Winter wrens.

**Figure 6:**
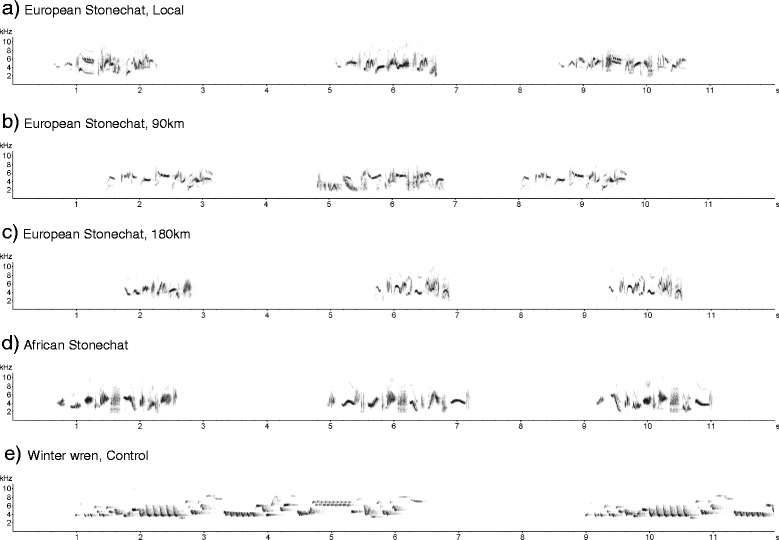
**Exemplary frequency spectrograms of acoustic stimuli used for the playback experiment.** Stimuli strings of European stonechat consisted of songs from members of **(a)** the local population and from populations at distances of **(b)** 90 km, and **(c)** 180 km from the study area. Furthermore, we played back song of **(d)** African stonechats, and **(e)** Winter wrens as a control.

We analysed the songs of all five stimuli origins (sampling frequency: 44.1 kHz; resolution: 16 bit, Figure 6) with the software Avisoft Sound Analysis Pro, version 5.1.09 (Raimund Specht, Berlin, Germany). We examined the song duration, number of elements per song, element rate (number of elements per second), minimum and maximum frequency, peak frequency (frequency of the highest amplitude sound), and bandwidth for all populations (Additional file 1: Figure S3). With the automatic parameter measurements setup, we obtained the minimum and maximum frequency values measuring at a standard decibel threshold (here −20 dB, total option) below the peak in the power spectra [70].

To assess the song repertoire size, we analysed 100 consecutive songs of each male Stonechat (n =20) from the local population. In Stonechats, a song typically consists of a sequence of motifs, and these in turn each contain several consecutive elements (Additional file 1: Figure S3). They are stereotypically repeated at a constant rate, and thereby distinguishable from all other song types. In general, song motifs, rather than complete songs, are shared within a population. The mean song repertoire consists of 16 ± 3.06 unique song types.

### Playback experiment

To reveal behavioural responsiveness of male and female Stonechats to songs of different dialects, we performed a field-based playback experiment by simulating a territorial intrusion with songs of distinct dialects during the egg laying or incubation stage (13^th^ – 26^th^ April, 2011). Each subject received five playback trials with the sequence of exposure determined by a randomized block design created by Randlist 1.2 (DatInf GmbH, Tübingen, Germany) to account for bias by trial order effects. Stimuli strings consisted of songs from the three European and the single African Stonechat populations. As a control, we used song of heterospecific Winter wrens, *Troglodytes hiemalis*. This species was chosen following the rationale by Grant and Grant [6], using a species that is similar in note structure and frequency range, but has never been heard by the tested birds.

To avoid inclusion of rare motifs, we selected song types with defined common motifs, which are shared between members of a population, and thus are representative of each population. However, this implies that the interpretation of song discrimination between populations should be taken with caution. The standardized stimuli selection of common and locally shared songs most likely excluded overlapping songs among populations. Their incorporation may have led to a slight decrease in the discrimination ability, and thus a potential overestimation of the responsiveness. To increase the number of independent samples, and thus improve the reliability and external validity, we generated a unique stimulus for each trial [71]. Each stimulus song was only used once for the entire study. We tested females and males simultaneously on their territories, and therefore each stimulus string was used once for both sexes (i.e. 28 × 5 = 140 unique playback stimuli). Each stimulus comprised song types from one individual’s recording following the natural syntax of Stonechat song. We used 25 unique songs in total for each stimulus string, which were filtered (1 kHz high-pass filter) and normalised in peak amplitude (i.e. the amplitude of each song was adjusted to 75 % of the maximum amplitude). Songs were divided by pauses of 4.5 seconds. A trial comprised all five population stimuli played back consecutively in a random order, each with a duration of 150 seconds. Each stimulus string was followed by at least 150 seconds of silence. To ascertain a comparable behavioural response of the latency to approach for each stimulus, playback strings only started when the focal bird (males =28, females =15) was at a distance of at least 10 m from the caller (longest silence between consecutive strings 285 s). Hence, each trial was performed on an individual bird for a period of about 25 minutes in total depending on the start times of the consecutive playback stimuli. Stimuli were broadcasted with the caller Foxpro Scorpion X1B (digital game caller, FOXPRO Inc. Lewistown, USA), which could be operated with a remote control. It was mounted on top of a bush in the central area of a territory such that it was widely audible. Response songs were recorded during the entire trial. However, acoustic responses to the playback were rare, and thus were not included in further analysis.

### Decoy experiment

We conducted a second experiment to test the responsiveness towards morphological traits by using a stuffed decoy simulating a territorial intrusion. During the nestling or fledgling stage we placed the decoy (male in full adult plumage protected by an inconspicuous cage) in the centre of respective territories for ten minutes in total for each trial. Decoy stimuli consisted of males from (a) local Stonechats, (b) European Stonechats from Austria [distance 1,000 km, 41], (c) African Stonechats from Kenya [distance 4,000 km, [68]], and as a control (d) European Robins, *Erithacus rubecula*. From extensive observations, we know that Stonechats aggressively chase off other small insectivorous passerines with similar feeding habits. European robins meet this criterion but their preference of deciduous wooded habitats limits their familiarity to Stonechats. To avoid pseudo-replication we randomly chose from five different decoys per stimulus for each trial. Each focal Stonechat (male =16, female =14) received all stimuli in a randomized and balanced order. We conducted each trial on a different date (5^th^-18^th^ May, 2011) during morning hours with two days pause between trials.

### Behavioural measurements

All behavioural responses were observed from a distance of about 30 m and were documented continuously by dictating to the Marantz PMD 661. To quantify behaviour, we used descriptors that are commonly used to measure responses to territorial intrusions and mate attraction [72], including studies in Stonechats [43]. Specifically, we measured the latency of a bird to approach the playback or decoy within 5 m; the time a bird spent within this 5 m zone; and the number of tail flips, which are defined as up- and downward movements of the entire tail and indicate agitation in Stonechats [65]. The descriptive statistics of all behavioural responses can be found in the supplements (Additional file 1: Table S4).

### Statistical analysis

All statistical analyses were performed with the software R v. 3.1.0 [73]. Tests were two-tailed and significance was accepted at α = 0.05. We used principal component analyses (PCA, R package *FactoMineR* [74]) to compare song traits between groups (with and without both control groups) and then tested the first principal component in a general linear model (LM, R package *lme4* [75]) to identify the relationship between song traits and geographic distance of song origins from the local population. The latencies to approach within 5 m to the different stimuli were analysed using mixed-effects cox models (survival model) fitted by maximum likelihood accounting for breeding stage, randomized trial order, date and time (coxme, R package *survival* [76]). Subjects were included as random intercepts to control for repeated measures. A Spearman’s correlation was run to determine the relationship of the behavioural response between paired females and males. For the time spent within 5 m we used a generalized linear mixed model with a beta distribution and stimulus as random factor using WinBUGS software 1.4 (GLMM, R package *R2WinBUGS* [77,78]). In WinBUGS we focussed exclusively on differences between stimuli. We defined stimulus as a random factor to compare paths of all stimuli, and thus correct for multiple testing. The response number of tail flips in males was analysed with a general linear mixed model fitted by maximum likelihood methods (LMMs, R package lme4 [75]) controlling for breeding stage, trial order, date and time. Subjects were included as random intercepts to control for repeated measures. Predictions from the general and generalized linear mixed models (Bayesian methods) were calculated as the median of their posterior distributions, and the 2.5 and 97.5% credible intervals (CI).

## Additional file

Additional file 1: Figure S1. Geographic variation in the song of European stonechats as quantified by principal component analysis. **Table S1** Song traits of three European Stonechat populations. (a) Factor loadings of the principal component analysis for seven song traits. (b) Results of a general linear model testing whether the first principal component (PC1) differed between songs from different locations. **Figure S2** Female and male behavioural responses. The figures show the latency to approach within 5 m of a stimulus in females and males during (a) playback experiments and (b) decoy experiments. **Table S2** Behavioural response between pairs. **Table S3** Correlation of behavioural responses between females and males during playback and decoy experiment. **Figure S3** Spectrogram of an exemplary song in European stonechats. Indicated are typical measured song traits of Stonechats, i.e. song duration, total number of elements, minimum and maximum frequency, and bandwidth. **Table S4** Descriptive statistics of behavioural responses for a) playback and b) decoy experiment. **Figure S4** Trial order for the latency to approach during the playback experiment in (a) males and (b) females, and during the decoy experiment in (c) males and (d) females.
