# Supplementary material for: Behavioural response of a migratory songbird to geographic variation in song and morphology
Source: Front Zool. 2014 Nov 28;11:85. doi: 10.1186/s12983-014-0085-6 (PMC4256809; doi:10.1186/s12983-014-0085-6)
Supplement: Additional file 1: Figure S1. — Geographic variation in the song of European stonechats as quantified by principal component analysis. Table S1 Song traits of three European Stonechat populations. (a) Factor loadings of the principal component analysis for seven song traits. (b) Results of a general linear model testing whether the first principal component (PC1) differed between songs from different locations. Figure S2 Female and male behavioural responses. The figures show the latency to approach within 5 m of a stimulus in females and males during (a) playback experiments and (b) decoy experiments. Table S2 Behavioural response between pairs. Table S3 Correlation of behavioural responses between females and males during playback and decoy experiment. Figure S3 Spectrogram of an exemplary song in European stonechats. Indicated are typical measured song traits of Stonechats, i.e. song duration, total number of elements, minimum and maximum frequency, and bandwidth. Table S4 Descriptive statistics of behavioural responses for a) playback and b) decoy experiment. Figure S4 Trial order for the latency to approach during the playback experiment in (a) males and (b) females, and during the decoy experiment in (c) males and (d) females. [file 12983_2014_85_MOESM1_ESM.pdf]

Additional file 1

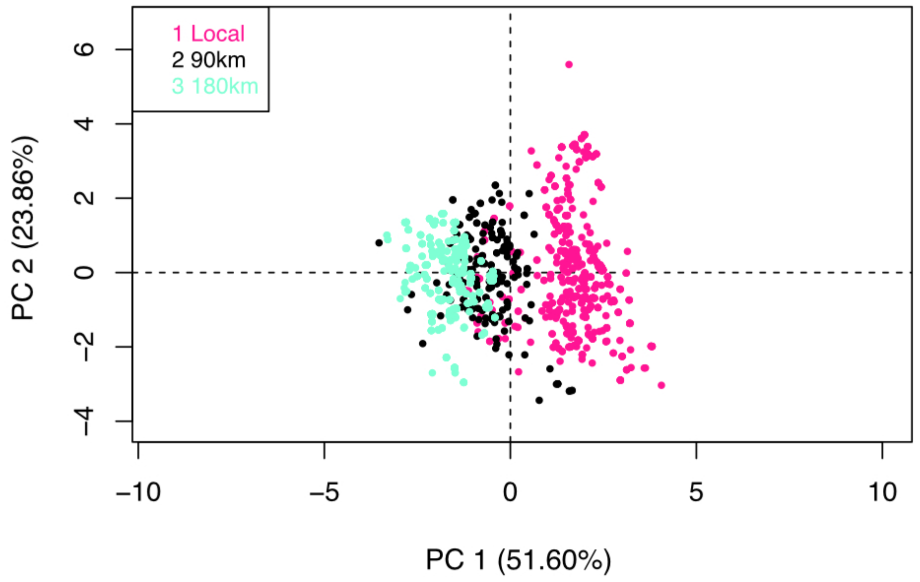

Figure S1 Geographic variation in the song of European stonechats as quantified by principal component analysis. Shown is (a) the variation in song structure of European Stonechats from (1) the local population, (2) a population from 90 km distance, and (3) from 180 km distance based on a principal component analysis (for details, see Table S1). Based on the first principal component, the song of the focal population differed significantly from the two neighbouring populations (90 and 180 km). The two latter populations did not differ significantly in their song from each other. However, differences in song traits reflected the geographic distances to the local population.

Table S1 Song traits of three European Stonechat populations. (a) Factor loadings of the principal component analysis for seven song traits of (1) the local population, (2) the population from 90 km distance, and (3) the population from 180 km distance. (b) Results of general linear model testing whether the first principal component (PC1) differed between songs from different locations, estimated by maximum likelihood methods. Estimates for the different song locations refer to differences from the intercept estimate, which represents song traits of the local population. Subjects were included as random intercepts to control for repeated measures. 'Significant' differences are shown in bold; n = 81.

(a)

	**PC 1**	**PC 2**	**PC 3**
song duration	0.31	0.53	0.05
no. of elements	-0.15	0.74	-0.06
element rate	0.39	-0.39	0.15
peak frequency	-0.02	0.10	0.96

	PC 1	PC 2	PC 3
min. frequency	0.49	0.01	0.09
max. frequency	-0.48	-0.13	0.19
bandwidth	-0.51	-0.06	0.03
eigenvalue	3.61	1.67	1.04
% variance	51.60	23.86	14.89

(b)

**fixed effects**	**estimate**	**s.e.m**	**t**	**p**
**intercept**	-0.04	0.11	**-0.35**	**0.73**
**90 km**	-1.02	0.15	**15.51**	**<0.001**
**180 km**	2.29	0.15	**-6.67**	**<0.001**

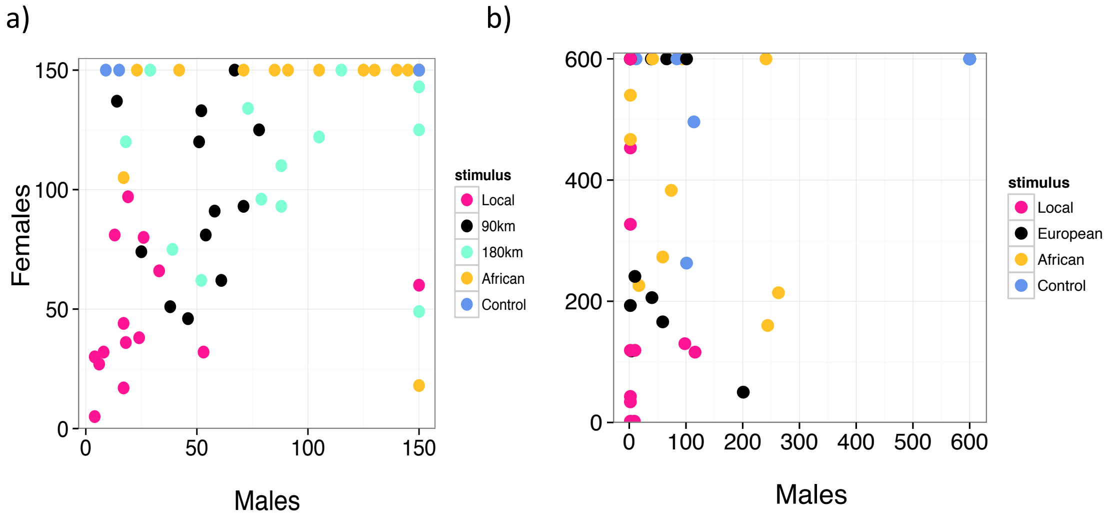

Figure S2 Female and male behavioural responses. The figures show the latency to approach within 5 m of a stimulus in females and males during (a) playback experiments in response to song of European Stonechats from (1) the local population, (2) the population from 90 km distance, (3) the population from 180 km distance, (4) African stonechats, and (5) control stimuli (Winter wren); and (b) decoy experiments in response to stimuli from (1) local, (2) European, (3) African, and (4) control stimuli (European Robin).

Table S2 Behavioural response between pairs

	estimate	hazard ratio	s.e.m.	z	p
**(a) playback**					
pairs	-0.07	0.93	0.14	-0.54	0.59
**sex (female vs. male)**	1.19	3.27	0.25	4.78	**<0.001**
breeding stage	-0.36	0.70	0.76	-0.48	0.63
trial order	0.07	1.07	0.16	0.41	0.68
date	0.06	1.06	0.16	0.36	0.72
time	-0.009	0.99	0.10	-0.01	0.99
**(b) decoy**					
pairs	-0.02	0.98	0.04	-0.39	0.70
**sex (female vs. male)**	1.71	5.52	0.29	5.88	**<0.001**
breeding stage	0.03	1.03	0.19	0.15	0.88
**trial order**	-0.36	0.70	0.17	-2.08	**0.04**
date	0.09	1.10	0.12	0.79	0.43
time	-0.01	0.99	0.01	-1.12	0.26

Results of the cox mixed-effects model with estimates, hazard ratio, standard error, z-value, and p-value fitted by maximum likelihood of the latency to approach controlling for sex, breeding stage, trial order, date and time. Subjects were included as random intercepts to control for repeated measures. ‘Significant’ differences are shown in bold.

Table S3 Correlation of behavioural responses between females and males during playback and decoy experiment

	rho	p	n
**playback**	0.51	<0.001	15
**decoy**	0.23	0.103	14

Results of Spearman’s rank correlation test for the latency to approach within 5 m of the stimulus between pair mates; data shows correlation coefficient rho, p-value, and sample size.

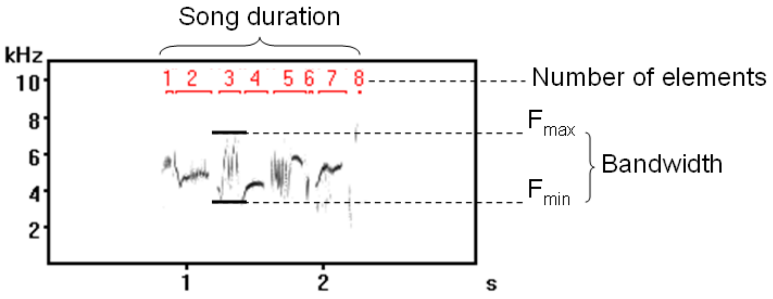

Figure S3 Spectrogram of an exemplary song in European stonechats. Indicated are typical measured song traits of Stonechats, i.e. song duration, total number of elements, minimum and maximum frequency, and bandwidth.

Table S4 Descriptive statistics of behavioural responses for a) playback (n = 28) and b) decoy (n = 16) experiment.

**stimulus**	**time spent within 5m (s)**	**number of tail flips**	**latency to approach (s)**	**latency to approach in females (s)**
**a) playback**				
**local**	117.36 ± 36.88	75.11 ± 47.38	22.21 ± 29.77	46.07 ± 26.71
**90 km**	92.07 ± 40.04	38.54 ± 32.10	50.54 ± 41.19	104.50 ± 37.85
**180 km**	70.79 ± 44.90	17.82 ± 19.88	73.71 ± 46.15	112.79 ± 33.47
**African**	63.54 ± 47.08	21.21 ± 25.06	83.50 ± 8.19	137.36 ± 36.39
**control**	19.88 ± 46.02	2.38 ± 4.60	115.83 ± 60.59	150.00 ± 0
**b) decoy**				
**local**	307.21 ± 223.89	121.36 ± 168.80	23.50 ± 40.79	212.80 ± 224.43
**European**	272.50 ± 229.62	111.93 ± 135.40	50.00 ± 65.40	381.17 ± 233.22
**African**	389.27 ±194.67	93.73 ± 109.52	69.80 ± 97.65	451.00 ± 175.40
**control**	314.67 ± 218.86	92.60 ± 134.66	460.70 ± 239.98	566.08 ± 95.49

Shown are the mean ± standard deviation of the time spent within 5 m, the number of tail flips and the latency to approach of males ($n_{playback}$ = 28, n_{decoy}=16) and females ($n_{playback}$ = 15, n_{decoy}=14) for (1) the local, (2) 90 km, (3) 180 km, (4) African, and (5) control stimuli.

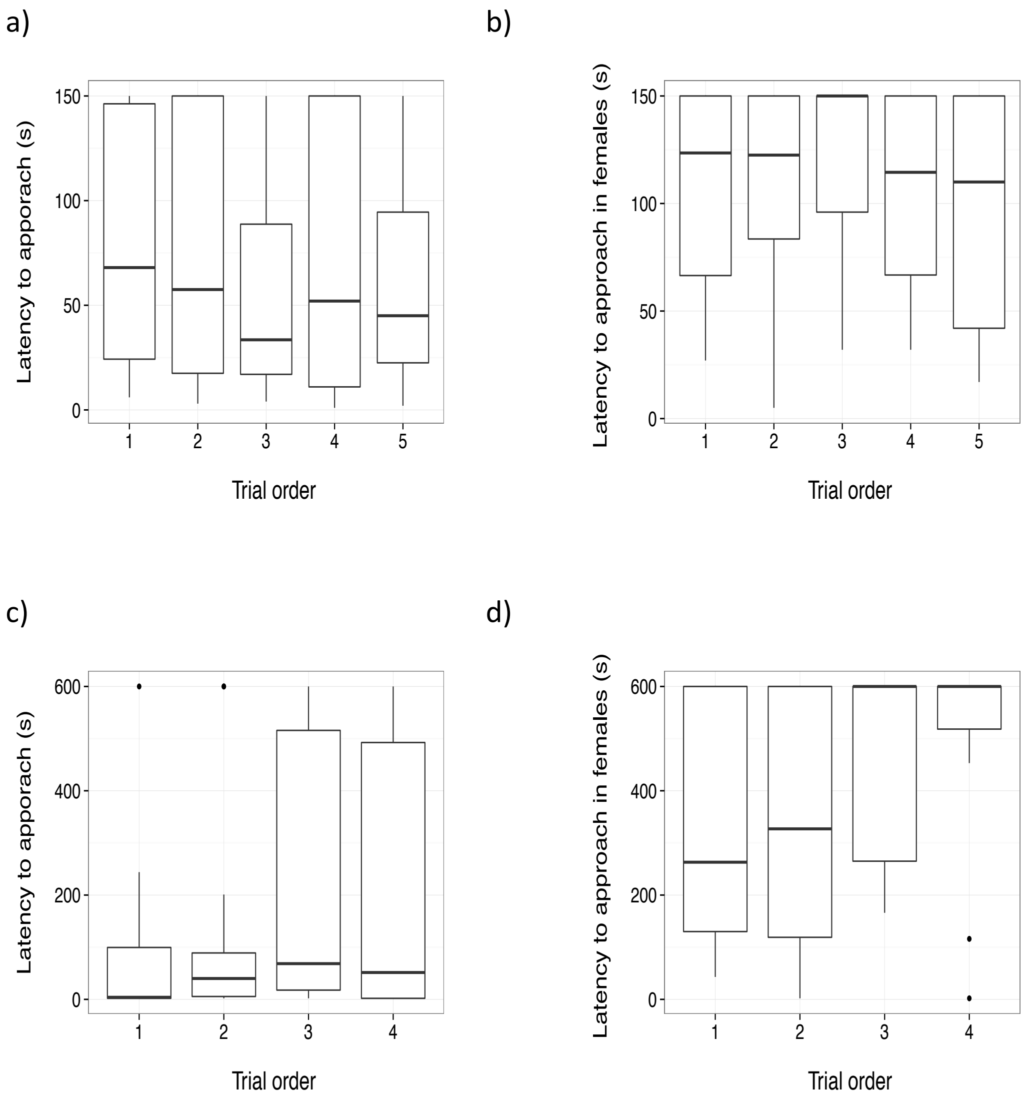

Figure S4 Trial order for the latency to approach during the playback experiment in (a) males and (b) females, and during the decoy experiment in (c) males and (d) females. Box plots represent, from bottom to top: minimum, lower quartile, median, upper quartile and maximum. Dots indicate observations further than one s.d. away from the mean.
